# Progress and Challenges in the Use of MAP1LC3 as a Legitimate Marker for Measuring Dynamic Autophagy In Vivo

**DOI:** 10.3390/cells9051321

**Published:** 2020-05-25

**Authors:** Srinivasa Reddy Bonam, Jagadeesh Bayry, Mario P. Tschan, Sylviane Muller

**Affiliations:** 1 CNRS, Biotechnology and Cell Signaling, Ecole Supérieure de Biotechnologie de Strasbourg, Illkirch, 67412 Strasbourg University/Laboratory of Excellence Medalis, 67000 Strasbourg, France; 2 Institut National de la Santé et de la Recherche Médicale, Centre de Recherche des Cordeliers, Sorbonne Université, Université de Paris, 75006 Paris, France; jagadeesh.bayry@crc.jussieu.fr; 3 Institute of Pathology, Division of Experimental Pathology, University of Bern, 3008 Bern, Switzerland; mario.tschan@pathology.unibe.ch; 4 Fédération Hospitalo-Universitaire OMICARE, Fédération de Médecine Translationnelle de Strasbourg, Strasbourg University, 67000 Strasbourg, France; 5 University of Strasbourg Institute for Advanced Study, 67000 Strasbourg, France

**Keywords:** autophagy, autophagic flux, MAP1LC3, lysosome, in vivo autophagy assays

## Abstract

Tremendous efforts have been made these last decades to increase our knowledge of intracellular degradative systems, especially in the field of autophagy. The role of autophagy in the maintenance of cell homeostasis is well documented and the existence of defects in the autophagic machinery has been largely described in diseases and aging. Determining the alterations occurring in the many forms of autophagy that coexist in cells and tissues remains complicated, as this cellular process is highly dynamic in nature and can vary from organ to organ in the same individual. Although autophagy is extensively studied, its functioning in different tissues and its links with other biological processes is still poorly understood. Several assays have been developed to monitor autophagy activity in vitro, ex vivo, and in vivo, based on different markers, the use of various inhibitors and activators, and distinct techniques. This review emphasizes the methods applied to measure (macro-)autophagy in tissue samples and in vivo via a protein, which centrally intervenes in the autophagy pathway, the microtubule-associated protein 1A/1B-light chain 3 (MAP1LC3), which is the most widely used marker and the first identified to associate with autophagosomal structures. These approaches are presented and discussed in terms of pros and cons. Some recommendations are provided to improve the reliability of the interpretation of results.

## 1. Introduction

Autophagy (from the ancient Greek “αὐτόφαγος”, meaning “self-eating”) is a critical component of cell homeostasis. This pivotal process contributes to the cellular quality control and energetic regulation of tissues via the lysosomal processing and recycling of vital cellular components [1]. Three main forms of autophagy are classically described that are macroautophagy, microautophagy, and chaperone-mediated autophagy (CMA), but in fact, there are many more forms and subtypes of each (as the different types of microautophagy, for example), working concomitantly in the cell to control its viability. Some of these processes are highly selective, some others are less, but all share a certain degree of selectivity to mediate the degradation of specific targets [2,3,4,5,6].

Much autophagic research has been originally done by exploiting organisms such as the versatile yeast and drosophila, and then followed by mammals. Over the past decades, significant advances have been made, leading to a better understanding of the molecular mechanisms driving autophagy in its many intricate physiological functions. These activities are thought to be particularly important in all aspects of cell life including differentiation and development, adaptation to metabolic stresses, degradation of dangerous cargo (e.g., misfolded or aggregated proteins, proteins produced daily in excess, damaged organelles, intracellular pathogens), and regulation of immune responses. It occurs, however, that these normally protective functions become harmful when autophagy processes are altered. Recent studies centered on the molecular biology of autophagy have especially addressed these issues in diseases as diverse as autoimmune and inflammatory syndromes, cancer, neurodegeneration, infectious diseases, cardiovascular disorders, metabolic diseases, intestinal bowel diseases, ischemia–reperfusion injury, and aging [5,7,8,9,10,11,12]. Mutations in numerous autophagy-related (*ATG*) genes have been linked to specific diseases, although rarely to one single pathology [4]. These mutations affect the proper functioning and regulation of autophagy and lysosomal activity, as well as cargo delivery. Their consequences also interfere in the crosstalk of autophagy with the many other forms of cellular circuits, such as apoptosis, necrosis, and ferroptosis [6].

These last years, the molecular elements of autophagy pathways characterizing macroautophagy, CMA, and more recently endosomal microautophagy (eMI), have been extensively studied. The cascade of events giving rise to the coordinated emergence of diversified organelles, the implication of multiple ATG genes, the identification of proteins complexes, for example, the ULK1 serine–threonine kinase (involving ULK1, FIP200, ATG13, and ATG101) and the VPS34/BECLIN-1 (BECN1) complex, which act sequentially in the autophagic processes, have been precisely examined [4,13,14,15,16]. These studies have permitted to pinpoint a number of markers that characterize different steps or forms of the autophagy process [17]. The combined use of drugs acting as activators or inhibitors on selected components and of genetically manipulated eukaryotic models have greatly contributed to the identification of successive steps and key components of the main autophagic pathways [14,18]. Particular attention should be given when certain drugs (e.g., bafilomycin A1) are used experimentally as their effect can critically depend on the time and concentration used as well as on the cell type. These drugs have also allowed demonstrating the potential interest of targeting autophagy for therapeutic applications [7,9,17]. Examples of such pharmacological agents and their main mechanism of action are listed in Table 1. Several comprehensive reviews extensively document their effects and some of their limitations [4,7,9,10,17,18,19,20,21,22].

Discovered in our team, a 21-mer phosphopeptide called P140, which primarily targets CMA in vitro and ex vivo and corrects altered macroautophagy occurring in inflammatory pathological contexts, showed its therapeutic effectiveness both in animal models of autoimmunity [26,27,28,29,30] and in patients with lupus [31,32]. Given to lupus mice with active disease, P140 reduced the overexpression level of lysosomal-associated membrane protein (LAMP)2A and HSPA8, two key players of CMA [33,34,35]. In vitro, P140 inhibited CMA in a cell line that stably expressed a CMA reporter [34,35]. In NOD.H-2h4 mice that develop a primary Sjögren’s-like syndrome, P140 rescued sick mice from some autophagy defects presumably associated with the ULK1 complex, and significantly reduced the formation of tertiary lymphoid structures in salivary glands, a hallmark characteristic of this disease in patients [30]. The expression of BECN1, which was raised in diseased NOD.H-2h4 mice, a feature that could explain, at least in part, the clinical and biological beneficial effect of P140 in this mouse model.

Another peptide, the cell-penetrating peptide Tat-BECN1, rather acts as an activator of autophagy [36]. It was found to exert protection against viral and bacterial infection both in vitro and in vivo, and some effectiveness in other pathological conditions such as heart failure in response to cardiac hypertrophy, for example [4]. The activity of some rationally-designed analogs of Tat-BECN1 peptide, like Tat-D11 (Novus Biologicals), prove significantly more potent than the original sequence in different assays and indications. A peptide named humanin initially identified from surviving neurons in patients with Alzheimer’s disease (AD), was described as a direct enhancer of CMA that acts by increasing substrate binding and translocation into lysosomes [37]. It interacts with HSP90 and stabilizes the binding of this chaperone to CMA cargos as they bind to the lysosomal membrane. It was shown that humanin exerts cardioprotective and neuroprotective properties in diseases such as AD, cardiovascular diseases, stroke, myocardial infarction, diabetes, and cancer. These peptides and other molecules that target specific autophagy elements represent potential therapeutics that might be successfully applied to treat patients affected by pathologies for which current drugs are clearly insufficient with severe secondary effects, or to individuals who are refractory to the existing medications.

When developing new chemical strategies of intervention that target autophagy, an important step is to demonstrate that in vivo, and not only in vitro or ex vivo, the molecule is effective on the expected autophagic target that has been identified in the upstream investigations. Indeed, in vivo, it may well be that the molecule induces its effect as thought, i.e., directly on the expected autophagy component but it might also act via an autophagy-independent mechanism or on an alternative metabolic system that in fine impact autophagy. It is effectively well known that numerous autophagy-related markers are also involved in other metabolic pathways. It is the case of BECN1, HSPA8, MTOR, and many others [4,23]. Yet, measuring autophagy in vivo or even in tissue samples (biopsies) collected from patients, for example, remains a challenge. Assessment of autophagic flux in whole organisms is even more challenging. Specific assays are limited and often remain non validated given the variety of tissues and organisms that are studied. The aim of this review is to gather useful information compiled from the existing literature and generated by our own experience, to analyze the validity of proposed methods and to discuss their potential and limitations. An emphasis is placed on microtubule-associated protein 1A/1B-light chain 3 (MAP1LC3) that is central in the autophagy process and especially in macroautophagy. Present at low and variable levels of expression in a normal situation, its distribution is widespread, for example in the bone marrow, brain, heart, placenta, thyroid, bladder, and several other organs and tissues.

## 2. The Microtubule-Associated Protein 1A/1B-Light Chain 3 (MAP1LC3)

**Figure 1:**
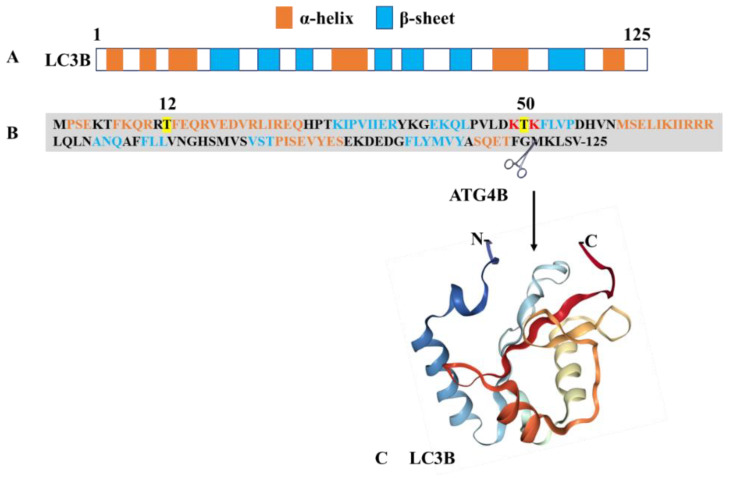
Structure of MAP1LC3B. The protein presents as a monomer with a molar mass of 13.6 kDa. (**A**) Pictorial illustration showing the alignment of α-helices and β-sheets in LC3B. (**B**) Primary structure of MAP1LC3B (NCBI reference ID: NP_073729.1) showing the positions of α-helixes and β-sheets. The sites of phosphorylation and acetylation are highlighted in yellow and red, respectively. The UbI domain is located at the C-terminus of the protein. (**C**) Three-dimensional (3D)-structure (PDB ID: 1V49) of the MAP1LC3B fragment 1–120. Figure generated using https://www.rcsb.org/, ref. doi:10.1093/bioinformatics/bty419.

Together with sequestosome (SQSTM1)/p62, MAP1LC3 is the most widely used marker that is followed experimentally to evaluate the extent of autophagy in cells. MAP1LC3 is a member of the highly conserved ATG8 protein family. It was initially described in the yeast (*S. cerevisiae*) and later identified in mammals [38,39]. MAP1LC3 belongs to a large family of seven members composed of MAP1LC3A, 3B, 3B2, 3C, and the γ-aminobutyric acid (GABA)-receptor-associated proteins (GABARAP, GABARAPL1, GABARAPL2) proteins. The NMR structure of MAP1LC3B (residues 1-120) has been determined (Figure 1) [40]. The members of this protein family share a common ubiquitin-like (Ubl) structures present at the C-terminus and two α-helices at their N-terminus, which are likely involved in protein–protein and lipid–protein interactions and post-translational modifications that occur during the autophagy process [41,42,43,44]. In the cytosol, newly synthesized MAP1LC3B is cleaved by the autophagy-related 4B cysteine peptidase (ATG4B) at the C-terminal glycine residues^120^ leading to MAP1LC3-I (Figure 1). Phosphatidylethanolamine (PE)-conjugation of MAP1LC3-I resulting in the lipidated MAP1LC3-II form requires ATG7 and ATG3, as well as the ATG12–ATG5–ATG16L1 complex [45,46,47]. Formation of the latter involves conjugation of ATG12 to ATG5, in a process mediated by ATG7 and ATG10, and further binding of the ATG12–ATG5 conjugate to an ATG16L1 dimer. In this process, ATG7 plays a central role as this E1-like activating enzyme enables both the lipidation of ATG8 proteins (MAP1LC3) and conjugation of ATG12 to ATG5. It is the only enzyme common to both conjugation pathways. At the isolation membrane and autophagosomes, MAP1LC3-II displays significant functions in the autophagy process, such as elongation, sealing isolation membranes, recognizing cargos, etc. [47]. The Ubl structures encompass β-strands with hydrophobic pockets, which are implicated in protein interactions. MAPL1LC3 is also present in the nucleus of a variety of cell types. In response to starvation, nuclear MAP1LC3 is deacetylated and trafficked out of the nucleus into the cytoplasm where it exerts its functions in autophagy. The protein is also enriched in nucleoli where it binds nuclear and nucleolar constituents, such as microtubule-associated protein 1B, tubulin, and ribosomal proteins.

MAP1LC3 proteins interact with many cofactors and ligands. All interacting proteins display a common ubiquitin-binding domain (UBD) and a short hydrophobic LC3-interacting region (LIR) motif that contains an N-terminal sequence W (tryptophan)xxL (leucine). The W residue is surrounded by other aromatic residues, tyrosine (Y) and phenylalanine (F), acidic residues, and sometimes serine (S) and threonine (T) residues to compose a consensus W/F/YXXL/I/V motif. These residues interact with the basic residues present in the Ubl domain of MAP1LC3 via electrostatic bridges (Figure 1). LIR motif-containing proteins encompass two hydrophobic residues, which are accommodated into the MAP1LC3 binding pockets [48]. The LIR motifs are post-translationally regulated and contribute to the different functions displayed by MAP1LC3 proteins, including selective autophagy process [48,49,50,51].

MAP1LC3 plays a key role in the autophagosome biogenesis machinery from the isolation membrane to the lysosomal stage. The recruitment of MAP1LC3 and other ATG proteins triggers vesicle expansion in a concerted manner and intervenes in the initial steps of membrane curvature. During this complex sequential process, MAP1LC3 is finely regulated by diverse post-translational modifications, which favor or on the contrary alter its activity. Thus, phosphorylation of MAP1LC3B at Thr^50^ by serine/threonine-protein kinases STK3/STK4 is essential for the autophagosome–lysosomal fusion process [52]. Phosphorylation at residue Ser^12^ by phosphokinase A (PKA) keeps MAP1LC3B unavailable for conjugation to PE and inhibits the autophagy process [53]. This modification is essential for phagophore expansion; its failure leads to defects in autophagosome formation. During stress or starvation conditions, PKA activity is inhibited and MAP1LC3B remains available for conjugation. On the other hand, acetylation regulates the MAP1LC3B translocation from the nucleus to the cytosol. The reason for the presence of LC3B in the nucleus remains an unanswered question. Acetylation at residues Lys^49^ and Lys^51^ makes the MAP1LC3B protein confined to the nucleus and not available for cytosolic ligases [54]. Of note, Lys^49^ and Lys^51^ form ionic interactions with ligands, especially with the LIR motifs. In stress conditions, especially starvation, deacetylation of MAP1LC3B by sirtuin 1 results in the availability of MAP1LC3B for ligases [54].

## 3. Current MAP1LC3-Based Methods Designed for Autophagy Research

**Figure 2:**
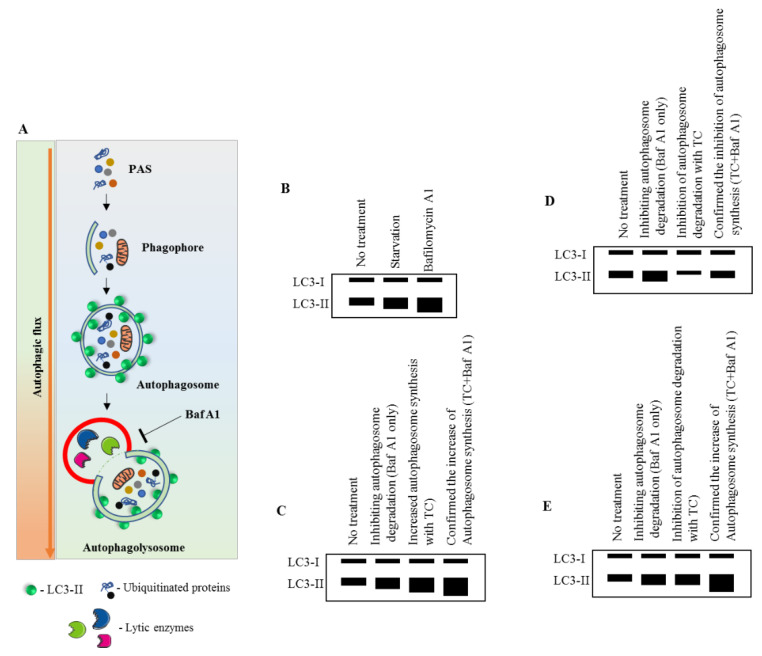
Immunoblot measurement of MAP1LC3B turnover and its dynamics. (**A**) Pictorial representation of the autophagic flux process. Baf A1 inhibits the autophagy process by blocking the autophagosome–lysosomal fusion. (**B**–**E**) Effect of a test compound on MAP1LC3-II expression. Without the use of Baf A1, it would be unpredictable to assess whether the compound is increasing the autophagosome synthesis or inhibiting the autophagosome degradation. Modified from [59]. See abbreviations in the abbreviations section.

**Figure 3:**
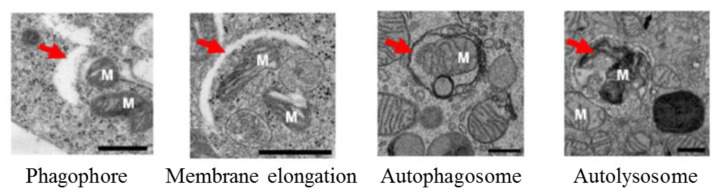
Macroautophagy process under electron microscopy. Red arrows are indicating the phagophore, membrane elongation, autophagosome, and autolysosome. M: mitochondria, scale bar: 500 nm. Adapted from [60].

MAP1LC3-based assays are widely used in current autophagy research [22,55,56,57,58]. In general, they offer the possibility to monitor the cellular autophagic activity in a routine manner using conventional laboratory equipment. A number of advantageous criteria, such as sensitivity, performance, specimen throughput, volume requirements, the limit of detection, ease of execution, instrument workspace, and the costs of equipment and disposal, are met. Commonly, the detection of processed MAP1LC3B-II by Western blot (Figure 2) or fluorescence studies, together with electron microscopy for autophagosome formation (Figure 3), is sufficient to qualify autophagy in cells.

These basic methods, applied to primary cells and cell lines, are often adequate for the first series of screening tests, but they may appear insufficient to further develop knowledge for therapeutic purposes. Indeed, they suffer each from intrinsic limitations that can introduce errors in the data analyses and survey results (Table 2). Furthermore, and most importantly, they often apply to cells and not to tissues and organs and are routinely performed in vitro/ex vivo and not in vivo.

In general, these methods have the capability for a high sample throughput and are used to sift large numbers of such samples for further application. As indicated, however, they also have some disadvantages and intrinsic limitations. The results that are generated to support possible changes in autophagy processes have also to be distinguished from effects induced by the many other forms of cell death [22,83].

## 4. MAP1LC3-Based Methods Designed for Studying Autophagy in Tissues

Several methods have been described to detect MAP1LC3 directly in fresh and fixed tissues collected from patients and experimental animal models. They are listed in Table 3 with their respective advantages and limitations (see also the Box 1).

Box 1 **Central aspects to consider while monitoring autophagy.**

General considerations

- Autophagy is a dynamic mechanism that requires flux measurements to be monitored.
- An increase of autophagy markers or factors detected at an early stage of the process may not give a clear idea on the entire process of autophagy as the blockade may appear at later stages. -Selection of mouse strain, while transfecting *MAP1LC3*, is a prominent factor in autophagy research, since for example, basal autophagy markedly differs between mouse strains.
- Detecting an increasing number of lysosomes may not reflect an increase in autophagic activity. LysoSensor may help to differentiate between the effects of autophagosome to lysosomes.
- It can be difficult to propose conclusions in some instances, especially in infections, in which the life cycle of pathogens affects the basal autophagic pathway.

Technical considerations

- Lack of highly specific autophagy inhibitors and activators remains a limiting factor.
- Autophagy-inducing agents, either for autophagy flux measurement or therapy, behave differently upon time and dosage.
- To evaluate novel molecules on inhibition or activation of autophagy, it is strongly recommended to use standard controls such as starved conditions and knockdown/knockout of key ATG proteins (e.g., *ATG3, 5, 7, 9a, 16L1, FIP200, AMBRA1, BECN1*) as a starting point.
- Autophagy modulator screening assays using GFP-MAP1LC3 benefit from secondary probes (GFP-MAP1LC3-RFP-LC3∆G, mRFP-GFP-MAP1LC3, mCherry-GFP-MAP1LC3) to clearly discriminate the effects on autophagosomes from effects on lysosomes or autolysosomes.

Specific considerations that apply when organs/tissue are studied

- The autophagic process (type, regulation, intensity) can differ from cell to cell, organ to organ/tissue in the same individual.
- Many fluorescence methods used in autophagy monitoring require specialized fixation methods, fresh tissue preparation, and rapid visualization. Most of the classical methods are not applicable in the case of the brain.

**Figure 4:**
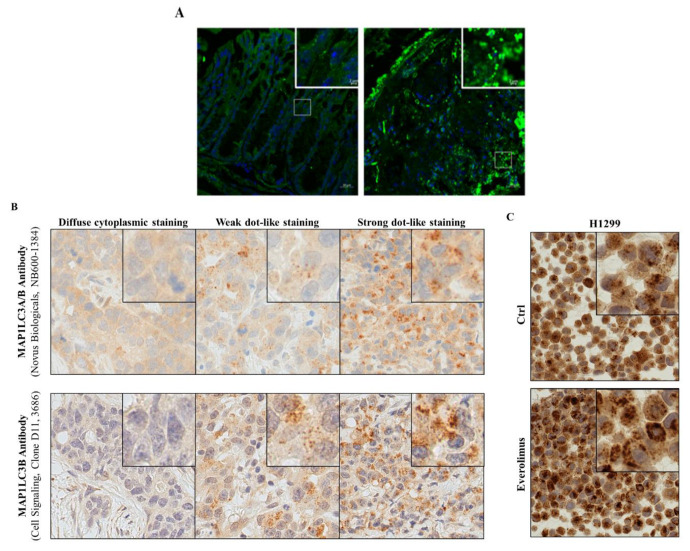
Immunofluorescence for MAP1LC3B. (**A**) Colon tissue sections of a control mouse and a mouse treated by TNBS to induce acute colitis. Staining for DNA with DAPI (blue) and for MAP1LC3 with Alexa-fluor labeled specific antibodies; (**B**) MAP1LC3A/B immunohistochemistry staining of three archived FFPE samples from patients with an adenocarcinoma of the lung. Different MAP1LC3A/B expression levels were visualized using two different antibodies, as indicated. (**C**) MAP1LC3A/B immunohistochemistry staining of FFPE-H1299 NSCLC cells that were either left untreated (Ctrl) or treated with everolimus, a selective inhibitor of MTOR activity used to stimulate autophagy. Detailed instructions on how the quantification of LC3B dot formation is done can be found in [84,86,87]. See abbreviations in the abbreviations section.

Examples are highlighted in Figure 4 using mouse colonic tissues, and human lung cancer tissues and cell lines.

A variety of suppliers provide adequate antibodies that should be evaluated and finely calibrated in each cell/tissue and methodological condition to gain an optimal signal/background ratio. A non-exhaustive list is given in Table 4.

As indicated, although MAP1LC3-II has been widely used as a marker to detect autophagy in various tissue samples from patients (biopsies or extraction of tissues at autopsy) or animal models, these assays cannot provide information on the dynamics of autophagy process and flux.

Detecting co-localization of key autophagy proteins or post-translational modifications associated with an active autophagy state might represent new approaches to better describe autophagic activity in tissue. In addition, the quality of the tissue materials from patients and animals, e.g., the time from removing the specimen to tissue fixation, is key to adequately assess autophagy. Clinical studies sometimes rely on quantifying MAP1LC3 staining intensity rather than analyzing MAP1LC3 dot formation to evaluate autophagic activity in archived patient samples. This is clearly less conclusive and should be avoided. Analyzing MAP1LC3 punctae formation, although not measuring the autophagic flux per se, better represents increased autophagic activity (Figure 4B,C) [87]. Improved and new techniques are critical to enabling autophagy flux analyses in the human tissues.

## 5. MAP1LC3-Based Methods Designed for Autophagy In Vivo

A number of approaches have been described to detect MAP1LC3 in living experimental animals. They are listed in Table 5 with respective performances and merits.

Despite the availability of various approaches for analyzing the autophagy in vivo in experimental models, and their potential use to screen therapeutic molecules and study the fundamental process of autophagy, their translation into the clinic remains limited. Further research is necessary to develop suitable probes that are safer and can be used in humans to analyze autophagy in vivo.

## 6. Conclusions

This short survey clearly identifies the crucial need for developing more performant assays permitting to screen autophagy regulator molecules in vivo. In view of the lack of suitable techniques to analyze autophagy flux in vivo in humans, the best possible solution would be to investigate autophagy flux by using cells derived from the affected organs/tissues of the patients with aberrations in the autophagy. Alternatively, induced pluripotent stem cell technology (3D tissue cultures or organoids) could be exploited in order to reprogram human somatic cells to obtain disease-relevant cell types for the investigations. Genome-wide studies have reported several disease-associated loci and genes that affect autophagy [4,91]. Though ex vivo, these cells provide an opportunity to investigate the role of autophagy in the pathogenesis of diseases and to get a clue on if therapeutic molecules that are deemed beneficial in the animal models could be used in the patients.

It is also important that MAP1LC3-based analyses need to be complemented with lysosomal function to confirm the intact autophagy process. Though MAP1LC3 analyses indicate the autophagy process, the functional intactness of lysosomes is critical to complete the process via degradation of macromolecules and damaged cellular components. In fact, several muscular disorders such as X-linked myopathy with excessive autophagy, inclusion body myositis (IBM), IBM-associated with Paget’s disease of the bone, frontotemporal dementia, and amyotrophic lateral sclerosis are associated with reduced proteolytic activity of lysosomes through enhanced autophagosome biogenesis was observed [92,93]. This feature has also been observed in lupus settings (Wang and Muller, unpublished data).

We deliberately focused this article on MAP1LC3-based analyses and macroautophagy. We listed the different ways of MAP1LC3 quantification in different settings and the arguments pro and against these measurements (see also Box 1 for recommendation). We emphasized here the importance of complementing MAP1LC3 autophagy measurements with nonMAP1LC3-based techniques. Other markers should be systematically followed such as SQSTM1, BECLIN-1, WIPI-1, ATG5/12, ATG14, ATG16L1, VPS34, LAMP2A, and others if possible, to support the results obtained with MAP1LC3. It is also important to distinguish the noncanonical autophagy process known as LC3-associated phagocytosis (LAP) from the canonical autophagy process. LAP and canonical autophagy processes do not require hierarchical intervention of all of the ATG proteins [94]. Both pathways do not play the same functions in cellular homeostasis and physiology, and therefore differentiating both processes can be highly relevant [95]. Recent studies on monocyte/macrophages revealed that LAP has a significant role in phenotype differentiation, particularly on the anti-inflammatory phenotype [96]. In addition to its role in regulating autoimmune response [97], LAP-mediated protective response against hepatic and systemic inflammation are also documented [98].

As reviewed in this article, biochemical assays, electron microscopy, and light microscopy are useful in assessing the autophagic process, however, these assays are cell type-, context-, and time-dependent. Immunologists or cell biologists who have equipped the laboratory with many techniques, should consider using as many markers possible and also, at least, two independent techniques to confirm the autophagic process. Autophagic flux analyses are especially recommended. Despite advancements in the methods to measure autophagy, there is still a lack of completeness, which should be answered with future developments. Novel methods, both in vitro and especially in vivo, to detect changes in the autophagy process in living tissues, are particularly awaited. They are decisive to discover and develop new, more effective drugs that target the autophagy machinery for patient care.

## Figures and Tables

**Table 1:** Examples of autophagic and lysosomal activators and inhibitors (non-exhaustive list) ^1^.

**Autophagy Activators**	**Mechanism of Action**
KU-0063794	Inhibits MTORC1 and MTORC2
Lithium, carbamezapine, sodium valproate	Reduces intracellular inositol levels by inhibiting their synthesis
Metformin	Activates AMPK
NVP-BEZ235	Inhibits PI3K/MTOR
Rapamycin, everolimus, temsirolimus (CCI-779), deforolimus, AZD8055	Interact wih FkBP-12 and the complex inhibits the activity of MTOR
Resveratrol	Activates SIRT1
Spermidine	Inhibits several acetyltransferases, e.g., EP300, IKI3, and SAS3
Statins (simvastatin)	Inhibits PKB/AKT
Sucrose	Impairs lysosomal function
Tamoxifen	Increases the intracellular level of ceramide. Abolishes the inhibitory effect of PI3K
TAT-Beclin-1	Inhibits the activity of PI3KC3 through competing of the binding of BECN1 to its negative regulator GAPR-1
Torkinib (PP242), torin 1	Inhibits MTORC1/2
Trehalose	Impairs lysosomal function
Tunicamycin	Induces ER stress in cells by inhibiting the first step of the biosynthesis of N-linked glycans in the proteins
**Autophagy Inhibitors**	**Mechanism of Action**
Azithromycin	Inhibits lysosomal v-ATPase and prevents the acidification process
Bafilomycin A1, concanamycin A	Both specifically bind to the V_0_ domain of v-ATPase and inhibit the intracellular pH gradients in endocytic and secretory organelles
CQ/HCQ	Increases the lysosomal pH (v-ATPase-independent)
Colchicine, Lys05, monensin, nigericine, nocodazole, SAHA (vorinostat), vinblastine	Inhibits autophagosome-lysosome fusion
E64d/pepstatin A	Inhibits cysteine and aspartic proteases
Leupeptin	Inhibits serine and cysteine proteases
NH_4_Cl	Increases the lysosomal pH (v-ATPase-independent)
P140 peptide	Inhibits the chaperone activity of HSPA8
Wortmannin, 3-methyladenine, LY294002	Inhibits class III PI3K

See abbreviations in the abbreviations section. ^1^ For more information, including their chemical structures, refer to the reviews [7,17,18,19,20,21,22,23,24,25].

**Table 2:** MAP1LC31-based standard assay for assessing autophagy and their limitations ^1^.

Techniques	Special Attention, Limitations and Pitfalls
Western blot assay (Figure 2)	Examining the expression of MAP1LC3-I and -II in the presence and absence of protease inhibitors is an absolute requirement ^2.^ Certain autophagosome-lysosome fusion competitors inhibit MTORC1, which initiates the induction of autophagy process [56,61,62]. The choice of inhibitors is decisive. For example, CQ can activate MAP1LC3-II formation independently from autophagy [63]. Specific detection of MAP1LC3-II is dependent on the type of antibody used. The majority of commercialized antibodies cross-react with several MAP1LC3 isoforms [64,65]. Antibodies may show different affinity for MAP1LC3-I and -II, and together with differing protein stability of the non- and lipidated forms western blotting bands require careful interpretation.
Fluorescence microscopy for detecting endogenous MAP1LC3	Discrimination between immatured, not yet closed and mature autophagosomes is required as both appear as punctate. Characterization of MAP1LC3 *puncta*. Advanced image analysis software’s (e.g., Top Hat algorithm of MetaMorph version 7.0 by Molecular Devices and G-Count by G-Angstrom) is a very useful tool to measure MAP1LC3 *puncta* [56]. Quantification can also be made manually by a trained and blinded observer. Discrimination of true autophagosomes devoid of MAP1LC3 aggregates, which are formed due to the aggregate prone proteins and autophagy-independent manner can be difficult.
Fluorescence microscopy for detecting reporters (e.g., GFP-MAP1LC3, mRFP-GFP-MAP1LC3, …)	Tissues from GFP-MAP1LC3 transgenic mice expresses more auto-fluorescence punctate structures [66]. Lack of GFP-MAP1LC3 expression in GFP-MAP1LC3 transgenic mice brain was observed, unlike other tissues. Cells deficient of ATG proteins, especially ATG5, would not generate MAP1LC3 punctate structures [67]. However, not all MAP1LC3 punctate structures are indicative of autophagy [58]. Loss of time-dependent fluorescence (GFP-MAP1LC3) intensity, but not mutant MAP1LC3, was observed [68]. In GFP- or mRFP-GFP-MAP1LC3 constructs, labelling may not give absolute results, especially if the pH of lysosomes is altered in pathological situations (as in lupus, for example, in which the mean lysosomal pH is raised [35]). Use of samples with or without inhibitors should be maintained for the better comparison (except for a few probes, e.g., GFP-MAP1LC3-RFP-MAP1LC3∆G). In terms of GFP-MAP1LC3-RFP-LC3∆G probe, more time (>2 h) is needed to observe significant changes in fluorescence ratio. Clone selection (transfection studies) should be monitored [69,70]. Assays based on the red fluorescent protein Keima cannot be used with fixed cells because the assay completely relies on lysosomal acidity [71].
Flow cytometry	Detects the different forms of endogenous MAP1LC3 (incl. MAP1LC3-I, MAP1LC3-II) proteins without any discrimination. Improved speed and statistical power when determining autophagic flux using tandem MAP1LC3 fusion proteins. Requires isolation of subcellular vesicles (e.g., autophagosomes, lysosomes) to highlight possible defects in the expression of endogenous MAP1LC3 protein levels [72]. Necessity to handle cell samples immediately [73].
Multispectral imaging flow cytometry	Combines features of flow cytometry with the imaging content of fluoresecent microscopy [74,75] Allows for detection of MAP1LC3 dot formation representative for MAP1LC3-II. Visualization of MAP1LC3 co-localization with lysosomal markers or other proteins.
Bioluminescence	Using a luminescent peptide to tag endo- and exogenous MAP1LC3 [76]. Allows easy detection and sensitive quantification of specific MAP1LC3 isoforms. Adapted to perform high throughput screening of compounds, for example. Small marker peptide allows for facilitated endogenous gene tagging using CRISPR/Cas9 technology. Does not allow detection of MAP1LC3 *punctae* formation.
MAP1LC3B time-resolved fluorescence transfer (TR-FRET) assay	Homogenous, mix-and-read assay that takes advantage of the required proximity of the donor and acceptor species for the generation of signal [77].
Electron microscopy (Figure 3)	Difficulty in discriminating the various types of vesicles (autolysosomes, endosomes, amphisomes, lysosomes) Difficulty to evaluate autophagy dynamics. No direct information obtained on lysosomal degradation. Time consuming. Technical errors, e.g., poor-fixation, sometimes leads to over or under looking observations [78]. Conventional methods, but not advanced electron microscopy methods, are not suitable to determine the volume and size of the inner cell compartments, due to the thin sections [60].
Long-lived protein degradation	Proteasome inhibitors should be used to specify the action of autophagy. Labelling efficiency is always a question, e.g., special culture media, without methionine, is required in non-radioactive labelling [79].
LDH sequestration assay	Lysosomal inhibitors always need to be used to measure autophagic flux [80].
Dextran sequestration assay	Loading fluorescently labelled dextran into cells is a delicate operation [81,82].

^1^ A few other conventional methods and/or nonspecific methods, such as isotope release from long-lived protein degradation (radiolabeling long-lived proteins with radioactive amino acid residues, such as [^14^C]-leucine, [^3^H]-leucine, [^14^C]-valine, or [^35^S]-methionine) and LDH assay (using LDH as a cargo) are not reviewed here as they are not routinely used. ^2^We are well aware that determining the ATG protein levels or the number of autophagosomes alone does not provide the overall estimation of autophagic activity since the process is very dynamic, and MAP1LC3-II can quickly degrade within the lysosomes. In fact, the two opposite scenarios, namely induction of autophagy or blockade in the downstream steps of autophagy leading to defective degradation, can result in an increased number of autophagosomes. Therefore, it is necessary to evaluate the autophagic flux by measuring the level of expression of MAP1LC3-I and -II in the presence of lysosomal inhibitors such as bafilomycin A1 or others. See abbreviations in the abbreviations section.

**Table 3:** MAP1LC3-based methods to measure autophagy in biopsies.

Method	Advantages	Limitations
Immunohistochemistry	High throughput analysis of MAP1LC3 localization in tissue arrays. Availability of fixed tissues in the clinic. Co-localization with additional autophagy-related proteins can be analyzed.	Availability of MAP1LC3 isoform specific antibodies with sufficient sensitivity for FFPE tissue sections. Quantifying MAP1LC3 *punctae* needs experienced pathologist.
Western blot analysis (from FFPE tissue) [84]	Distinction between MAP1LC3-I and -II.	A lot of tissue is needed to extract enough protein. Requires protein extraction from a cell mixture. Isolation of pure cell populations from the tissues would be needed to analyze cell-specific levels of MAP1LC3 expression. No information on MAP1LC3 localization.
In-situ hybridization [85]	Highly specific for MAP1LC3 isoforms. Allows to assess MAP1LC3 isoform expression levels in different cell types.	MAP1LC3 mRNA expression is not a “marker” of autophagy activity *per se*. One needs to assume that MAP1LC3 mRNA levels correlate with protein expression.

See abbreviations in the abbreviations section.

**Table 4:** Non-exhaustive list of MAP1LC3 antibodies.

Antibodies	Applications, Conditions ^1^	Limitations
Polyclonal anti-MAP1LC3B antibody (#NB600-1384, Novus Biologicals)	Western blot 1:1000 in 5% (*w*/*v*) non-fat milk, 3h, room temperature. For example, ref [84,86,87]	MAP1LC3A cross-reaction
Polyclonal affinity-purified rabbit anti-MAP1LC3B antibody (#2775; Cell signaling Technology)	Western blot, 1:1000 Used with human primary cells (PBMCs, dendritic cells, monocytes, macrophages), cell lines. For example ref [12]	
Rabbit anti-MAP1LC3B antibody (#3868,Cell Signaling)	Immunofluorescence 1:200 in PBS-1% (*w*/*v*) BSA.	
Anti-MAP1LC3B antibody (#M186-3, MBL Inc.)	Western blot 0.5 µg/mL and immunofluorescence 5 µg/mL in TBS containing 1% non-fat milk. Used for example with sciatic nerves sections from Lewis rats; with malignant glioblastoma-derived U-251MG cells; with mouse spleen and salivary glands cells; with colonic cells from mice with colitis (Figure 4). For example, ref. [29,30,57]	
Monoclonal rabbit anti-MAP1LC3B antibody, clone D11 (#3686, Cell Signaling)	Immunofluorescence. For example ref [84,86,87]	See the legend of Figure 4B
Polyclonal affinity-purified rabbit anti-MAP1LC3A/B antibody (#4108; Cell signaling Technology)	Immunofluorescence 1:200 dilution. Used with cell lines. For example, ref [12]	

^1^ These conditions are routinely used in our respective laboratories. They have to be adapted to each type of substrate and technique. See abbreviations in the abbreviations section.

**Table 5:** Methods to measure autophagy in vivo ^1,2^.

Method	Advantages	Limitations
Transgenic mice expressing GFP-MAP1LC3 and fluorescence microscopy	Allow the formation of autophagosomes to be studied. Used to study macroautophagy and mitophagy.	Do not permit the formation of autolysosome to be studied as GFP loses its fluorescence at acidic pH in lysosomes. Other transgenic mice are required [88] No measurement of autophagic flux. Cells possess auto-fluorescent punctate structures such as lipofuscin that is detectable in the green spectra. Always compare to non-transgenic control littermates [66].
mCherry-GFP-MAP1LC3 and mRFP-GFP-MAP1LC3 mouse and fluorescence microscopy	High time resolution.	Technical difficulty in distinguishing RFP/GFP double-positive and single positive *punctae*. Lack of performance to measure the basal autophagic flux.
GFP-MAP1LC3-RFP-LC3∆G mRNA (injected in animal eggs) and fluorescence microscopy	Measure the basal (low) and induced autophagy flux in embryos and tissues of zebrafish and mice [70].	

^1^ SBI-0206965 (Adooq Bioscience), a potent and selective inhibitor of ULK1, can be used to inhibit autophagy in vivo; is given intraperitoneally into mice at 2 mg/kg body weight in DMSO, once per day for 7 days [89]; other autophagy blockers can be used in vivo as control, e.g., CQ/HCQ, NH_4_Cl, bafilomycin A1 (in certain strict conditions), colchicine, vinblastine, and the inhibitor of lysosomal enzymes leupeptide [66]. ^2^ Measurement of chaperone-mediated autophagy (CMA) activity in vivo has been described [90]. See abbreviations in the abbreviations section.

## Glossary

**Amphisomes**	are single-membrane compartment issue from the fusion of autophagosomes and endosomes
**Autolysosomes**	are single-membered acidic vesicle formed by the fusion of autophagosome and lysosome
**Autophagosomes**	are well discriminated double-membrane vesicles in which intracellular material (incl. organelles and fragments of organelles) are sequestered and delivered to the lysosome for degradation
**Endosomes**	are single-membrane compartment formed by the process of endocytosis
**Lysosomes**	are digestive organelles of the cell, which bear an acidic environment (pH 4.5 to 5.0) to degrade material via different processes
**v-ATPases**	are membrane-associated proton pumps, which maintain the acidic environment in organelles, such as the endosomes, lysosomes, trans-Golgi network, and secretory granules.
**Abbreviations:**	
AMPK	adenosine monophosphate-activated protein kinase
ATG	autophagy-related
ATP	adenosine triphosphate
Baf A1	bafilomycin A1
BSA	bovine serum albumin
LC3-I/-II	MAP1LC3-I/-II
CQ/HCQ	chloroquine/hydroxychloroquine
Ctrl	control nonsmall
DAPI	4′,6-diamidino-2-phenylindole
DMSO	dimethyl sulfoxide
ER	endoplasmic reticulum
FFPE	formalin-fixed paraffin-embedded
FkBP-12	FK506 binding protein-12 kDa
GAPR-1	Golgi-associated pathogenesis related-1 protein
GFP	green fluorescent protein
GFP-MAP1LC3-RFP-LC3∆G	construct in which the C-terminus of GFP-LC3 is fused to RFP-LC3 protein deleted from its C-terminal glycine
HSP	heat shock protein
LDH	lactate dehydrogenase
MAP1LC3	microtubule-associated protein 1A/1B-light chain 3
mRFP	monomeric red fluorescent protein
MTOR	mechanistic target of rapamycin
MTORC1	mammalian target of rapamycin complex 1
NSCLC	nonsmall cell lung cancer
PBMCs	peripheral blood mononuclear cells
PBS	phosphate-buffered saline
PI3K	phosphatidylinositol 3-kinase
PKB/AKT	protein kinase B
RFP	red fluorescent protein
SAHA	suberanilohydroxamic acid
SIRT1	sirtuin 1
TBS	Tris-buffered saline
TC	trial compound
TNBS	2,4,6 trinitrobenzene sulfonic acid
v-ATPase	vacuolar-type H^+^-ATPase
